# Adjudicative efficacy of *Bifidobacterium animalis* subsp. *lactis* BLa80 in treating acute diarrhea in children: a randomized, double-blinded, placebo-controlled study

**DOI:** 10.1038/s41430-024-01428-6

**Published:** 2024-03-11

**Authors:** Ke Chen, Shanshan Jin, Yu Ma, Limei Cai, Ping Xu, Yang Nie, Li Luo, Qinghua Yu, Yang Shen, Zengyuan Zhou, Changqi Liu

**Affiliations:** 1 Department of Nutrition, Chengdu Women’s and Children’s Central Hospital, School of Medicine, University of Electronic Science and Technology of China, Chengdu, China; 2 Department of Neonatology, Dayi Maternal and Child Health Care Hospital, Chengdu, China; 3 Department of Neonatology, Qingbaijiang Maternal and Child Health Care Hospital, Chengdu, China; 4 Department of Child Health Care, Qingbaijiang Maternal and Child Health Care Hospital, Chengdu, China; 5 Department of Child Health Care, Chongzhou Maternal and Child Health Care Hospital, Chengdu, China; 6 Department of Pediatrics, Dayi Maternal and Child Health Care Hospital, Chengdu, China; 7 Laboratory of Microbiology, Immunology and Metabolism, Diprobio (Shanghai) Co., Limited, Shanghai, China; 8 School of Exercise and Nutritional Sciences, San Diego State University, San Diego, CA USA

**Keywords:** Randomized controlled trials, Paediatrics

## Abstract

The goal of this study is to assess the efficacy and safety of *Bifidobacterium animalis* subsp. *lactis* BLa80, as an adjunct treatment for diarrhea in children with a randomized, double-blinded, placebo-controlled study design. Eligible diarrheal children, aged 0–3 years without the need for antibiotic treatment based on clinical diagnosis when recruited, were randomized into the intervention group (IG, *n* = 58, with probiotic) or the control group (CG, *n* = 53, placebo). The primary assessment was the duration of diarrhea. Fecal samples were collected for biochemical index measurement, analysis of gut microbiome composition, and prediction of gene family abundances. The total duration of diarrhea in the IG (122.6 ± 13.1 h) was significantly shorter than in the CG (148.4 ± 17.6 h, *p* < 0.001). More children in the IG showed improvements in diarrhea compared to the CG, both in intention-to-treat analysis (81.7% vs. 40.0%, *p* < 0.001) and per protocol analysis (84.4% vs 45.3%, *p* < 0.001). Cathelicidin level in the IG was significantly higher than that in the CG after the intervention (4415.00 ± 1036.93 pg/g vs. 3679.49 ± 871.18 pg/g, *p* = 0.0175). The intervention led to an increased abundance of *Bifidobacterium breve* and *Collinsella aerofaciens* species, higher alpha-diversity (*p* < 0.05), and enrichment of functional genes in the gut microbiota related to immunity regulation. Administration of BLa80 at a dose of 5 × 10^9^ CFU/day resulted in a shorter duration of diarrhea and alterations in gut microbiome composition and gene functions.

## Introduction

Diarrhea is a common and recurrent disease in children, posing risks of malnutrition, restricted growth and development, and even fatality, especially in developing countries [1]. Numerous randomized, controlled trials have demonstrated the anti-diarrheal effects of specific probiotic strains, particularly in children [2–5]. The European Society for Pediatric Gastroenterology, Hepatology, and Nutrition and the European Society of Pediatric Infectious Diseases Expert Working Group stated that only probiotic strains with proven clinical efficacy and in appropriate dosage may be recommended as an adjuvant to treat acute gastroenteritis in children [6]. Currently, a wide array of probiotic products are available in the market, differing in excipients, microbial strains, amounts, and activity [7–10].

*Bifidobacterium animalis* subsp. *lactis* BLa80 is a distinctive strain isolated from human breast milk samples in the highland pastoral areas of Hongyuan, Sichuan Province, China, with independent intellectual property rights. The strain has been assigned a preservation number of CGMCC No. 22547 by the China General Microbiological Culture Collection Center (CGMCC). BLa80 exhibits strong adhesion and colonization abilities in the intestinal tract, which can decrease the incidence of diarrhea and enhance intestinal immunity in experimental mice [11]. The BLa80 strain can also increase the abundance of *bifidobacteria* and *lactobacillus* in human intestine [12]. Animal studies have found that BLa80 can improve stool frequency, weight, and water content, shorten intestinal transportation time, increase levels of acetic acid, propionic acid, and butyric acid in the intestine, and regulate the intestinal microbiota [13].

To our knowledge, no study has investigated the role of BLa80 in regulating gastrointestinal health in children with diarrhea. Therefore, the purpose of this research is to study the adjunctive clinical efficacy of the BLa80 strain in the treatment of acute watery diarrhea in children.

## Materials and methods

### Subjects and ethical approval

This is a multi-center, parallel randomized, controlled, double-blinded clinical intervention. Children of both sexes and aged 0–3 years who were outpatients and/or hospitalized with diarrhea were recruited between Dec. 2021 and Sep. 2022.

### Inclusion, exclusion, and withdrawal criteria

Diagnostic criteria for watery diarrhea: Increased fecal frequency (≥4 times/day) [14] with watery feces (Bristol fecal score above type 6).

Inclusion criteria:

1. Age: children 0–3 years old.
2. Duration of diarrhea (the time of the previous bowel movement, before the stool consistency had returned to normal, was used as the endpoint of the course of diarrhea): more than 12 h and less than 72 h;
3. No need for antibiotic treatment based on clinical diagnosis during recruitment;

Exclusion criteria:

1. Nervous system dysplasia and severe organic diseases;
2. Moderate and severe dehydration, serious diarrhea requiring Pediatric Intensive Care Unit (PICU) treatment, bloody stools;
3. The same probiotics taken within one month before the diagnosis of this illness;
4. Children are expected to receive antibiotic treatment during the trial.

Withdrawal criteria:

1. Children without any clinical records for evaluation;
2. Children taking drugs prohibited by the study, including hormones, immunosuppressive drugs, other probiotics, etc., during the treatment.

### Allocation sequence generation and concealment

To ensure the randomization of participants in the study, a research staff, independent of the study, generated an allocation sequence. This sequence outlined how participants were assigned to different interventions and was created using the RAND function in Excel. Subsequently, another research staff, unaffiliated with the study, executed the central remote telephone randomization process. This step was implemented to prevent children’s guardians and trial personnel from knowing the forthcoming allocations until after the recruitment was confirmed. Children meeting the inclusion criteria were coded with random numbers and assigned into one of two groups, each consisting of 60 randomly assigned children.

### Grouping and intervention

Recruited children were managed per the WHO guidelines [15]. Children in the intervention group (IG) received oral probiotics in addition to standard diarrhea management. The probiotic was given as a single sachet (Wecare Probiotics Co., Ltd., Production No.: SC10632050900407) containing BLa80 strain 5 × 10^9^ CFU/sachet and was taken daily for seven consecutive days starting on the first day of clinical treatment. Children in the control group (CG) underwent standard therapy and were given a reference sachet (placebo) containing only maltodextrin. The probiotic and placebo had similar appearance, taste, and smell and were provided in identical sachets with identical labelling, expect for the subject-specific randomization number. The children’s parents and/or guardians, clinicians, laboratory personnel, data managers, and statisticians remained blinded to group assignments until the end of data analysis.

### Data collection

After enrollment, the study staff performed assessments, recorded data on the clinical record form (CRF), and collected laboratory samples according to the protocol. During the trial period, the data of hospitalized children were recorded by the field workers. For discharged or outpatient children, parents took daily pictures of the child’s feces and sent them to the researcher for objective records. Fecal pictures were collected daily to confirm fecal type and evaluate treatment efficacy. Clinicians used the CRF to record the incidence of abdominal cramps, nausea, vomiting, fever, constipation, and low appetite in children during the treatment. The average daily Bristol fecal score was defined as the sum of the daily Bristol fecal score divided by the fecal frequency on a given day.

### Fecal immune and inflammation biomarkers assessment

Fecal samples were collected from all children before and after the intervention to measure levels of sIgA, calprotectin, human beta-defensin 2 (HBD-2), and cathelicidin (LL-37) using commercial enzyme-linked immunosorbent assay kits (Shanghai Enzyme-linked Biotechnology Co., Ltd./mlbio).

### Fecal microbiome analysis

A total of 158 fecal samples were collected for gut microbiome analyses, including 82 samples from 41 children in the IG before and after the intervention and 76 samples from 38 children in the CG. Genomic DNA from the samples was extracted using the CTAB/SDS method with the QIAamp Fast DNA fecal Mini Kit (Qiagen, Valencia, California, USA) according to the manufacturer’s instructions.

The isolated genomic DNA targeting the bacterial 16S rRNA gene V3–V4 region was amplified using the TransGen AP221-02 Kit (TransGen, Beijing, China). The library was sequenced on an Illumina NovaSeq platform, generating 250 bp paired-end reads. QIIME (Version 1.9.1) was used for calculating both alpha- (within sample) and beta- (between sample) diversity. Shannon, Simpson, Chao1, and ACE indices were used as indicators of the alpha diversity. Principal coordinate analysis (PCoA) based on Bray-Curtis distance was used to analyze β-diversity. Differential enrichment of the gut microbiome was analyzed using linear discriminant analysis effect size (LEfSe). To explore the functional profiles of the gut microbiome, Phylogenetic Investigation of Communities by Reconstruction of Unobserved States (PICRUSt) was performed based on 16S information [16].

### Efficacy judgement

According to the national pediatric diarrhea efficacy evaluation standards and similar studies [5, 14], the efficacy was assessed as follows:

Marked effectiveness: after 72 h of treatment, the frequency of diarrhea decreased to ≤2 times/day, and the fecal consistency and clinical manifestations returned to normal;

Normal Effectiveness: after 72 h of treatment, the frequency of diarrhea is reduced to ≥3, and <4 times/day, the fecal consistency is significantly reduced, and the clinical symptoms are basically disappeared;

Ineffectiveness: diarrhea without remission after treatment for 72 h;

Total efficiency = (number of marked effectiveness cases + number of normal effectiveness cases)/total number of cases × 100%.

### Statistical analysis

All efficacy analyses were performed on both the intention-to-treat (ITT) dataset and the per-protocol dataset, comprising participants who adhered to the protocol, completed the clinical intervention, and provided all necessary demographic and clinical data, especially information on changes in stool consistency and frequency. SAS version 9.2 for Windows (SAS Institute Inc., Cary, NC, USA) was used for all analyses.

*T* test was used to compare normally distributed data. Wilcoxon rank-sum test was used for data without a normal distribution. *χ*^2^ test was used to compare differences in treatment efficacy between the two groups for countable data. The frequency of feces and average daily Bristol fecal score between the two groups before and after the intervention were compared using repeated measures analysis of variance (ANOVA). A *p* value less than 0.05 was considered statistically significant.

### Sample size

In a previous study on the treatment of rotavirus enteritis using three combined strains [5], the duration of diarrhea in the CG and the IG was reported as 143.9 ± 19.8 h and 121.4 ± 13.7 h, respectively (a reduction of nearly 24 h). With *β* = 0.8, *α* = 0.05 (bilateral), the sample size for each group was calculated to be 50 subjects. Accounting for a 20% dropout rate, we selected a sample size of 120 subjects with 60 subjects in each group.

## Result

### Basic clinical and demographic data

A total of 120 children were enrolled and randomized into the study, all of whom were included in the ITT analysis. Among them, 60 were assigned to the IG and 60 to the CG. Nine children were excluded from the PP analysis due to major protocol deviations. All allocated children who received at least one dose of study product were included in the ITT analysis, resulting in a total of 110 infants in the PP dataset (58 in the IG and 53 in the CG). Figure l depicts a flowchart illustrating participant involvement. There was no significant difference in demographics and clinical data before the intervention between the two groups (*p* > 0.05, supplementary material-1, SM-1). There were no children worsened to the extent of requiring admission to PICU during the treatment.

**Fig. 1:**
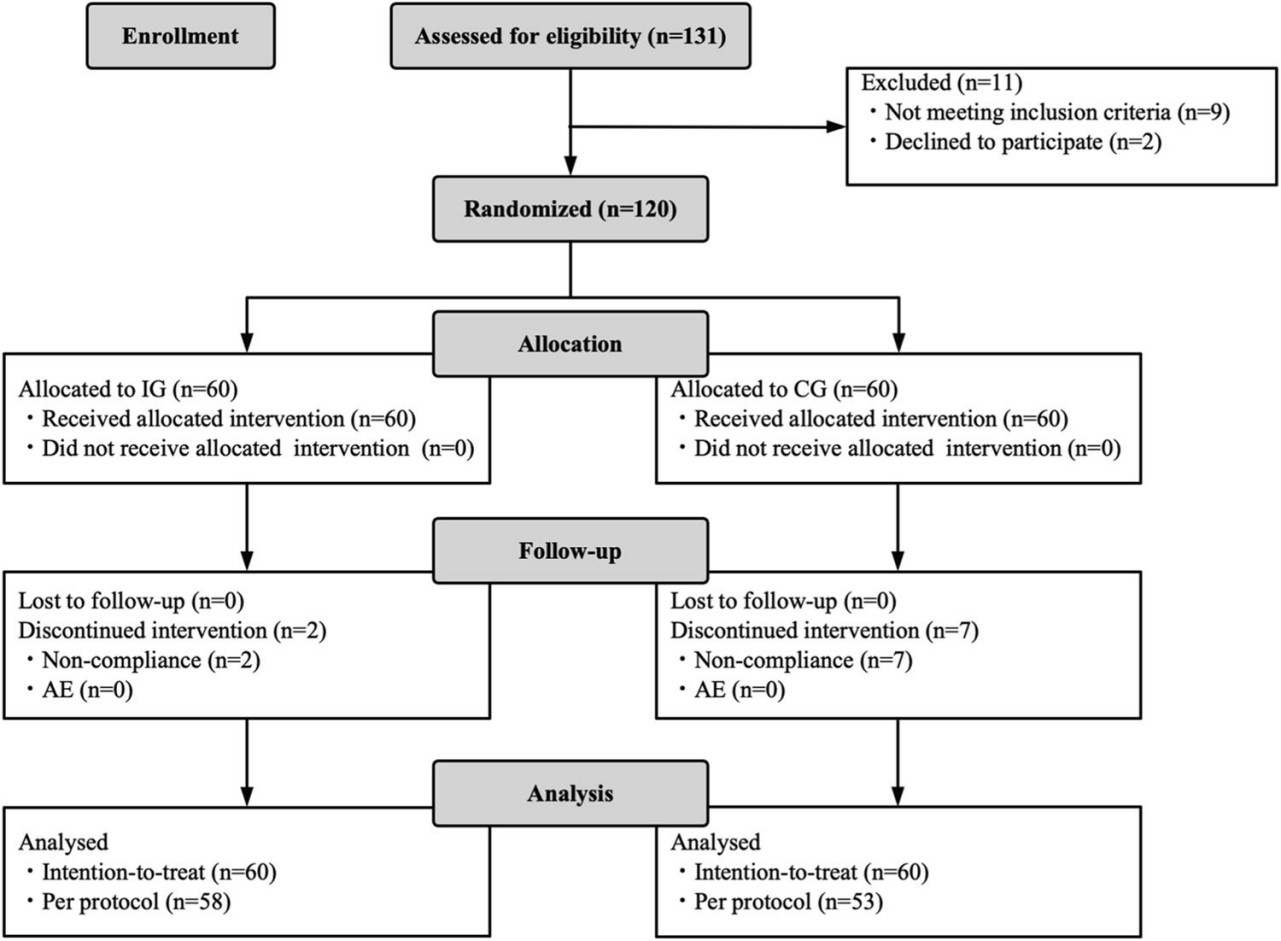
Flowchart of subject enrollment and study progress. IG intervention group, CG control group, AE adverse events.

### Efficiency of probiotic intervention on diarrhea

After the intervention, the duration of diarrhea in children from the IG (122.6 ± 13.1 h) was significantly shorter (*p* < 0.001) compared to the CG (148.4 ± 17.6 h) (Table 1).

**Table 1:** Efficiency of probiotic intervention on diarrhea [*n* (%)].

Items		IG (*n* = 60)	CG (*n* = 60)	RR (95%CI)	*χ*^2^ values	*p* values
No. of marked efficiency [*n* (%)]^a^		31 (51.7)	10 (16.7)	0.099 (0.037, 0.263)	24.55	<0.001
No. of normal efficiency [*n* (%)]^a^		18 (30.0)	14 (23.3)	0.238 (0.090, 0.628)		
No. of inefficiency [*n* (%)]^a^		11 (18.3)	36 (60.0)	1.000		
No. of total efficiency [*n* (%)]^a^		49 (81.7)	24 (40.0)	0.150 (0.065, 0.344)	21.86	<0.001
Total duration of diarrhea (hours)^ab^	mean ± SD	122.6 ± 13.1	148.4 ± 17.6	—	9.11	<0.001
	median (P25, P75)	124 (46, 144)	150 (48, 172)	—		

*IG* intervention group, *CG* control group, *SD* standard deviation, *RR* relative risk, *CI* confidence interval.

^a^Compared with the CG, the difference was statistically significant (*p* < 0.05).

^b^The time of the previous bowel movement before the stool consistency had returned to normal was used as the endpoint of the course of diarrhea.

After a 72 h treatment, the IG exhibited a marked effective rate of 51.7% (31/60), a normal effective rate of 30.0% (18/60), and a total effective rate of 81.7% (49/60). In contrast, the CG showed rates of 16.7% (10/60), 23.3% (14/60), and 40.0% (24/60), respectively, for the ITT dataset. The marked and total effective rates in the IG were significantly higher than those in the CG (*p* < 0.05, Table 1). The PP analysis also showed a significantly higher total effective rate in the IG compared to the CG [84.4% (49/58) vs. 45.3% (24/53), *χ*^2^ = 18.90, *p* < 0.001].

### Efficiency of probiotic intervention on daily fecal frequency

**Fig. 2:**
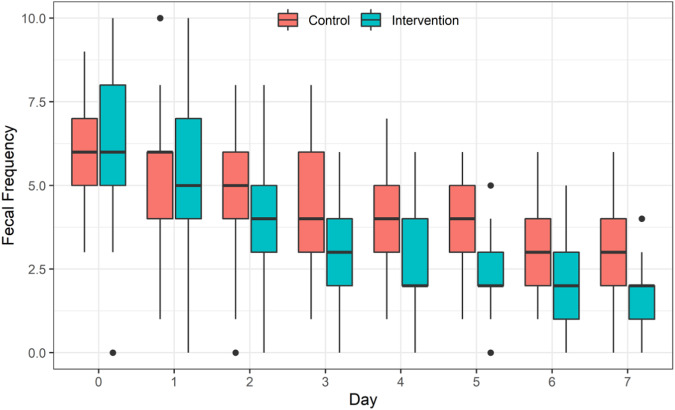
Efficiency of probiotic intervention on daily fecal frequency of children between two groups. The box represents quartiles and the thick line inside indicates the median. Whiskers extend 1.5 times the interquartile range from the first and third quartiles. Any data beyond the whiskers is depicted with dots. There was a significant difference in the daily frequency of feces between the intervention group (IG) and the control group (CG). The frequency of feces in the IG was significantly less than that in the CG (*F* = 202.84, *p* < 0.001).

Standard diarrhea treatment led to a significant reduction in daily frequency of feces within each group (*F* = 230.45, *p* < 0.001). Moreover, a notable difference in the daily frequency of feces was observed between the IG and the CG. The frequency of feces in the IG was significantly lower compared to the CG (*F* = 202.84 *p* < 0.001). Additionally, a significant interaction was noted between standard treatment and the use of probiotics (*F* = 6.39, *p* < 0.001) (Fig. 2, SM-2 for data).

### Efficiency of probiotic intervention on the average of daily Bristol fecal score

**Fig. 3:**
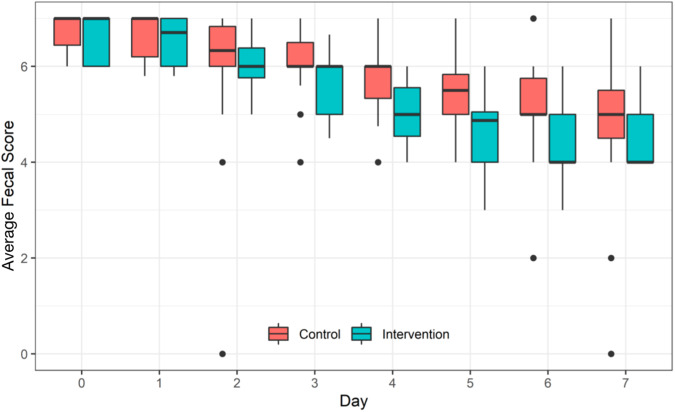
Efficiency of probiotic intervention on the average of daily Bristol fecal score of children between the two groups by repeated measures analysis of variance. Children in the intervention group had significantly lower average of daily Bristol fecal score than children in the control group (*F* = 15.81, *p* < 0.001).

The repeated measures ANOVA indicated a significant decrease in the average daily Bristol fecal score of children in both groups with the standard treatment (*F* = 241.58, *p* < 0.001). Children in the IG had a significantly lower average daily Bristol fecal score than those in the CG (*F* = 15.81, *p* < 0.001). Furthermore, a significant interaction was observed between standard treatment and the use of probiotics (*F* = 6.87, *p* < 0.001) (Fig. 3, SM-3 for data).

### Efficiency of probiotic intervention on fecal immune and inflammation biomarkers

**Fig. 4:**
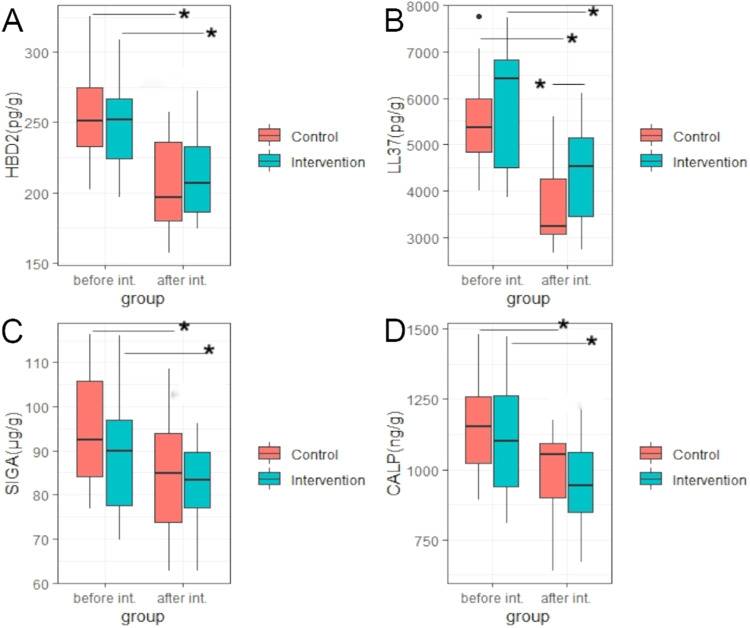
Efficiency of probiotic intervention on fecal biochemical indices of children. *, difference with statistical significance by *t* test within or between two groups; **A** HBD-2, human beta-defensin -2; **B** LL37, cathelicidin ; **C** SIGA, sIgA; **D** CALP calprotectin, int Intervention; Intervention, IG group; Control, CG group.

After the intervention, there was a significant decrease in all immune and inflammation biomarkers in both groups (all *p* values < 0.05). The intervention led to a significant increase in LL-37 levels in children (4415.00 ± 1036.93 pg/g vs. 3679.49 ± 871.18 pg/g for IG and CG children, respectively, *p* = 0.0175), while there were no changes in slgA, calprotectin, and HBD-2 levels (all *p* values > 0.05) (Fig. 4, SM-4 for data).

### Efficiency of probiotic intervention on fecal gut microbiota

**Fig. 5:**
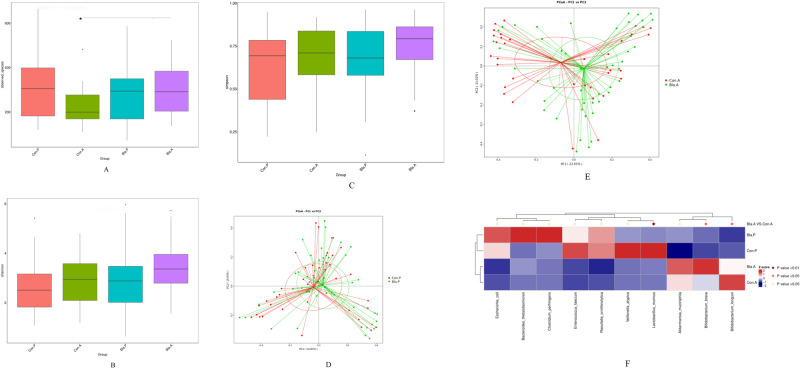
Effects of intervention on gut microbiota. **A**, **B** and **C** Effects of probiotic intervention on alpha diversity indices of the gut microbiota between the two groups before and after the intervention.*, significant difference between the two groups by *t* test; **A** number of observed species; **B** Simpson index; **C** Shannon index. **D**, **E** Analysis of the beta diversity calculated on the Principal coordinate analysis (PCoA) based on Bray–Curtis distance. **D** before intervention; **E** after intervention. **F** The MetaStat Complex Heat map showing the differential abundance at species level between the two groups with statistical significance by *t*-test. Con.A, CG after intervention; Con.P, CG before intervention; Bla.A, IG after intervention; Bla.P, IG before intervention.

As shown in Fig. 5, the analysis of alpha diversity revealed that the richness estimates (calculated in observed species) were significantly higher in the IG compared to the CG after the intervention (*p* < 0.05). However, no significant differences were observed in the Shannon and Simpson indices between the groups after the intervention (all *p* values > 0.05). There were also no significant differences in these three indices between the two groups before the intervention (all *p* values > 0.05) (Fig. 5A–C).

The PCoA plot based on Bray-Curtis distance showed that axis 1 (PC1) explained 14.83% of the variability and axis 2 (PC2) explained 10.93% of the variability before the intervention. The samples of children in IG and CG were spatially very close to each other. However, after the intervention, the samples from the two groups showed a trending spatial separation (Fig. 5D, E).

Furthermore, the MetaStat method confirmed that the abundance of *Bifidobacterium brevein* and *Lactobacillus murinus* in the IG was significantly higher than that in the CG, while the abundance of *Bifidobacterium longum* was significantly lower in the IG (*p* < 0.05) (Fig. 5F).

LEfSe analysis identified 8 taxa that were differentially abundant between the two groups before the intervention. In comparison to the CG, the BLa80 treatment increased the abundance of one family (*Coriobacteriaceae*), one order (*Coriobacteriales*), and one class (*Coriobacteriia*), while one family (*Streptococcaceae*) was enriched in the CG. Furthermore, LDA scores (>4.0) derived from the LEfSe analysis at the genus and species levels identified several bacterial genera and species that differed in the two groups. Notable high abundance in the *Collinsella* genus and *Collinsella aerofaciens* species were found in children from the IG, while the children in the CG were enriched with the *Streptococcus* genera after the intervention (SM 5).

### BLa80 treatment changed the functional gene composition of gut microbiota

The PICRUSt result showed a notable difference in functional genes within the gut microbiome before and after BLa80 treatment, suggesting a potential impact on the metabolic pathways of the gut microbiome. The proportion of 108 sub-functional genes in the gut microbiome exhibited significant changes after BLa80 treatment only, with top 30 means in each group displayed (All comparisons before and after Bla80 intervention demonstrated statistical significance, with corresponding *p*-values provided in SM 6). After BLa80 treatment, certain functional genes, such as DNA repair and recombination protein, purine metabolism, ribosome, peptidases, pyrimidine metabolism, chromosome, ribosome biogenesis, and amino acid related enzymes, were enriched. Conversely, other genes, such as ABC transports, general function prediction only, transcription factors, two-component system, were reduced. The results indicated a significant influence of BLa80 treatment on the composition of functional genes in the gut microbiome.

### Incidence of probiotic intervention related adverse reactions during treatment

No incidence of abdominal colic, nausea, vomiting, fever, low appetite, or other symptoms related to the probiotic and placebo interventions was observed in both groups.

## Discussion

Overall, this RCT study provided initial evidence that the administration of BLa80 at a daily dose of 5 × 10^9^ CFU for seven days, while demonstrating limited clinical significance, statistically shortened the course of diarrhea, improved clinical treatment efficiency, and induced alterations in gut microbiome composition.

### Efficiency of probiotic intervention on diarrhea, fecal consistency, and frequency

Replenishing fluids and electrolytes have been shown to have limited impact on reducing the frequency and duration of diarrhea [17]. Several meta-analyses have demonstrated that the use of specific probiotics can be effective in reducing the duration of diarrhea and improving clinical outcomes for patients [18–20].

In this study, we found that compared to children in the CG, the use of *ready-to-eat* probiotic powder (BLa80) for one week significantly improved the therapeutic efficacy for diarrhea. Notably, the beneficial adjuvant effect of BLa80 in treating acute watery diarrhea was evident as early as the second day of the treatment, consistent with findings from a similar probiotic intervention [21]. The reduced fecal frequency can be attributed to the combined effects of standard diarrhea treatment, probiotic intervention, and their interaction. The probiotic intervention exhibited additional treatment effects in alleviating diarrhea after excluding the time effect. Moreover, the interaction effect indicated that, in conjunction with standard treatment, the probiotic intervention group experienced a more efficient reduction in fecal frequency.

Besides the reduced stool frequency, the probiotic significantly improved the consistency of feces. By the 4th day after the intervention, watery feces transformed into type 5 soft feces in the IG, whereas mushy feces persisted in the CG until the 7th day after intervention.

### The efficiency of probiotic intervention on fecal immune and inflammation biomarkers

The changes observed in immunity biomarkers in this study are different from findings in other studies using different strains [22, 23]. The decreased HBD-2, LL-37, calprotectin, and sIgA levels after the treatment in both groups may be attributed to several factors. Some studies [24–26] have shown that immune and inflammation biomarkers are significantly increased to fight against the dominant or potential inflammation of the intestine. This is confirmed by high levels of these biomarkers observed before the intervention in our study. The swift improvement in all infants’ intestinal symptoms led to the anticipated decline in immune and inflammation biomarkers. Additionally, the initial sample size was calculated based on diarrhea duration. The sample size for fecal analysis might be insufficient to distinguish significant differences between the two groups after the intervention, except for LL-37 indicator, which might be more sensitive than others. Furthermore, the change in calprotectin [27, 28], which reflects the inflammatory state of the intestine, can be similarly explained as the alterations in immune and inflammation biomarkers.

### Efficiency of probiotic intervention on gut microbiota

Recent studies on the use of *Bifidobacterium animalis* as an adjuvant therapy for gastrointestinal diseases in both children and adults have shown its potential to influence the intestinal micro-ecological composition and exhibit a synergistic effect in improving prognosis [22, 29–31]. Consistent with these findings, the present study demonstrates that BLa80 administration can increase richness estimates of alpha diversity and change gut microbiome composition.

The observed species-based richness estimates of intestinal microbiota in the IG after BLa80 administration surpassed those in the CG. PCoA further revealed distinct microbial community composition between the two groups after the intervention. Considering the inherent high diversity of the gut microbiome in infants following probiotic administration and the associated decrease in the risk of gastrointestinal diseases due to the enhanced variety and abundance of dominant or potentially beneficial bacteria, the high diversity of gut microbiome after BLa80 use might be a manifestation of the microbiota-regulating effects of BLa80.

According to functional gene prediction analysis, BLa80 treatment enriched the functional genes involved in purine metabolism of the gut microbiome. Extracellular purines play a pivotal role [32] in controlling the chemotaxis, activation, proliferation, and differentiation of immune cells. This suggests that the enriched purine metabolism may contribute to the amelioration of diarrhea by modulating intestinal immunity. Moreover, a study [33] has demonstrated that ribosome biogenesis unexpectedly regulates dsDNA-sensing to restrict virus reproduction and regulate inflammation. Consequently, the enriched functional gene expression related to ribosome biogenesis may also be connected to the clinical manifestation of diarrhea to some extent. The same speculation [34] can also be applied to the enriched functional gene predictions associated with DNA repair and recombination protein and peptidases [35] after BLa80 treatment. However, it is crucial to note that these hypotheses, including the reduced gene expression, require further well-designed animal and in vitro experiments for confirmation, in order to understand the exact mechanism of action of BLa80.

#### Limitation analysis

Firstly, the present study did not identify possible pathogens responsible for the watery diarrhea, limiting the ability to investigate different responses of specific pathogens to BLa80. Secondly, the use of a single dose of BLa80 at 5 × 10^9^ CFU/day precluded an exploration of the optimal dose-response relationship. Thirdly, the trial spanned only one week, preventing the observation of possible long-term effects of BLa80 on children’s health and gut microbiome. Lastly, dietary habits of the participants were not assessed.

## Conclusions

To conclude, we did not observe any adverse effects of the BLa80 intervention during our study period, affirming its safety for infants. The administration of the *Bifidobacterium animalis* subsp. *lactis* BLa80 at a dose of 5 × 10^9^ CFU/day to children aged 0–3 years resulted in shorter duration of diarrhea, faster improvement in fecal consistency, and alterations in gut microbiome.

### Supplementary information

supplementary materials

## Data Availability

The data supporting the findings of this study are available from the corresponding author on upon reasonable request. Raw 16S rRNA gene sequence for all feces samples used in this study have been deposited in the National Center for Biotechnology Information BioProject database with the BioProject ID PRJNA1068284.
