# Supplementary material for: Adjudicative efficacy of Bifidobacterium animalis subsp. lactis BLa80 in treating acute diarrhea in children: a randomized, double-blinded, placebo-controlled study
Source: Eur J Clin Nutr. 2024 Mar 11;78(6):501–8. doi: 10.1038/s41430-024-01428-6 (PMC11182741; doi:10.1038/s41430-024-01428-6)
Supplement: Supplementary file 1 — supplementary materials [file 41430_2024_1428_MOESM1_ESM.docx]

Supplementary material-1

Table Basic clinical and demographic data between two groups before intervention [mean ± standard deviation or median (P25, p75)]

| Items | | | | IG | CG | *χ^2^* values | *p* values |
| --- | --- | --- | --- | --- | --- | --- | --- |
| No. | | | | 58 | 53 | - | - |
| Sex composition [male, n (%)]^*^ | | | | 23(39.7) | 30(56.7) | 3.189 | 0.074 |
| Age (m)^*^ | | mean ± SD | | 15.74±18.53 | 10.48±15.25 | 1.813 | 0.178^+^ |
|  |  | median (P25, P75) | | 1.9(0.63, 34.37) | 1.23(0.47, 21.97) |  |  |
| Patients source | Out-patient | | | 52 | 50 | 0.816 | 0.366 |
|  | In-patient | | | 6 | 3 |  |  |
| Full term or not [yes, n (%)]^*^ | | | | 56(96.6) | 49(92.5) | - | 0.381^#^ |
| Delivery mode [vaginal, n (%)]^*^ | | | | 36(62.1) | 27(50.9) | 1.397 | 0.237 |
| Slight dehydration [yes, n (%)] | | | | 0(0) | 1(1.9) | - | 0.477^#^ |
| Registered residence [urban, n (%)]^*^ | | | | 52(89.7) | 48(90.6) | - | 0.755^#^ |
| Family history of allergic disease [yes, n (%)] | | | | 0 | 0 | - | - |
| Previous allergic disease [yes, n (%)] | | | | 0 | 0 | - | - |
| Sum of daily  Bristol fecal score one day before intervention^*^ | | | mean ± SD | 39.57±12.01 | 42.53±10.75 | 1.967 | 0.161^+^ |
|  |  |  | median (P25, P75) | 38.5(30.0, 49.0) | 42（35,49） |  |  |
| Daily fecal frequency one day before intervention^*^ | | | mean ± SD | 5.91±1.68 | 6.34±1.49 | 1.922 | 0.166^+^ |
|  |  |  | median (P25, P75) | 6(5, 7) | 6（5,7） |  |  |
| Mean of daily Bristol fecal score one day before intervention^*^ | | | mean ± SD | 6.66±0.45 | 6.70±0.42 | 0.028 | 0.868^+^ |
|  |  |  | median (P25, P75) | 7(6, 7) | 7（6.4,7） |  |  |

*, There was no significant difference between the IG and the CG (*p*>0.05); #, Fisher exact probability method; +, Wilcoxon non-parametric test between groups for data with skewed distribution and uneven variance; IG, intervention group; CG, control group; SD, standard deviation.

Supplementary material-2

Table Efficiency of probiotic intervention on daily fecal frequency of children between two groups [mean ± standard deviation, median (P25, p75)]

| Daily fecal frequency | | | IG (n=58) | CG (n=53) |
| --- | --- | --- | --- | --- |
| one day before intervention | | mean ± SD | 5.91±1.68 | 6.34±1.49 |
|  |  | median (P25, P75) | 6(5, 7) | 6（5, 7） |
| 1st day during intervention | | mean ± SD | 5.28±1.58 | 5.58±1.84 |
|  |  | median (P25, P75) | 5(4, 6) | 6（4, 6） |
| 2nd day during intervention | | mean ± SD | 4.12±1.46 | 4.68±1.85 |
|  | | median (P25, P75) | 4(3, 5) | 5（4, 6） |
| 3rd day during intervention | | mean ± SD | 3.41±1.28 | 4.38±1.78 |
|  | | median (P25, P75) | 3(3, 4) | 4（3, 6） |
| 4th day during intervention | | mean ± SD | 2.55±1.20 | 3.87±1.47 |
|  | | median (P25, P75) | 2(2, 3) | 4（3, 5） |
| 5th day during intervention | | mean ± SD | 2.05±1.10 | 3.63±1.38 |
|  | | median (P25, P75) | 2(1, 2) | 4（3, 5） |
| 6th day during intervention | | mean ± SD | 1.83±0.92 | 3.17±1.27 |
|  | | median (P25, P75) | 2(1, 2) | 3（2, 4） |
| 7th day during intervention | | mean ± SD | 1.59±0.90 | 2.75±1.36 |
|  | | median (P25, P75) | 1(1, 2) | 3（2, 4） |
| Time efficiency | *F* value^*^ | 230.45 | | |
|  | *p* value | < 0.001 | | |
| Intervention efficiency | *F* value^*^ | 202.84 | | |
|  | *p* value | < 0.001 | | |
| Time-intervention interaction efficiency | *F* value^*^ | 6.39 | | |
|  | *p* value | < 0.001^**^ | | |

*, analysis of variance of repeated measurement data; IG, intervention group; CG, control group; SD, standard deviation; **, post-hoc analysis.

Supplementary material-3

Table Efficiency of probiotic intervention on the mean of daily Bristol fecal score of children between the two groups [mean ± standard deviation, median (P25, p75)]

| Mean of daily Bristol fecal score | | | IG (n=58) | CG (n=53) |
| --- | --- | --- | --- | --- |
| One day before intervention | | mean ± SD | 6.66±0.45 | 6.70±0.42 |
|  |  | median (P25, P75) | 7(6, 7) | 7（6.4,7） |
| 1st day during intervention | | mean ± SD | 6.55±0.45 | 6.66±0.44 |
|  |  | median (P25, P75) | 6.71(6, 7) | 7（6.2,7） |
| 2nd day during intervention | | mean ± SD | 5.97±0.62 | 6.20±1.08 |
|  | | median (P25, P75) | 6(5.75, 6.40) | 6.33（6,6.83） |
| 3rd day during intervention | | mean ± SD | 5.66±0.55 | 6.02±0.62 |
|  | | median (P25, P75) | 6(5, 6) | 6（6,6.5） |
| 4th day during intervention | | mean ± SD | 5.01±0.67 | 5.68±0.72 |
|  | | median (P25, P75) | 5(4.5,5.57) | 6（5.3,6） |
| 5th day during intervention | | mean ± SD | 4.68±0.75 | 5.39±0.68 |
|  | | median (P25, P75) | 4.88(4, 5.1) | 5.5（5.0,5.83） |
| 6th day during intervention | | mean ± SD | 4.52±0.69 | 5.17±0.76 |
|  | | median (P25, P75) | 4(4, 5) | 5（5,5.75） |
| 7th day during intervention | | mean ± SD | 4.50±0.61 | 5.00±0.75 |
|  | | median (P25, P75) | 4(4, 5) | 5（5,5.5） |
| Time efficiency | *F* value* | 241.58 | | |
|  | *p* value | < 0.001 | | |
| Intervention efficiency | *F* value* | 15.81 | | |
|  | *p* value | < 0.001 | | |
| Time－intervention interaction efficiency | *F* value* | 6.87 | | |
|  | *p* value | < 0.001^**^ | | |

*, analysis of variance of repeated measurement data; IG, intervention group; CG, control group; SD, standard deviation; **, post-hoc analysis.

Supplementary material-4

**Table** Efficiency of intervention on fecal biochemical indexes of children between the two groups [mean ± standard deviation, median (P25, p75)]

| Indexes |  | IG (n=25) | CG (n=19) | *t* values*^*^* | *p* values*^*^* |
| --- | --- | --- | --- | --- | --- |
| fecal human HBD-2 level (pg/g) | | | | | |
| Baseline | median (P25, P75) | 251.76(224.01, 267.27) | 250.98(232.82, 284.04) |  |  |
|  | mean ± SD | 251.77±31.46 | 254.72±34.73 | 0.29 | 0.7695 |
| After intervention | median (P25, P75) | 206.71(185.12, 233.15) | 196.60(169.95, 241.11) |  |  |
|  | mean ± SD | 211.16±28.51 | 206.40±34.30 | 0.50 | 0.6213 |
|  |  | *t*=4.78, *p*<0.001^#^ | *t*=4.315, *p*<0.001^#^ |  |  |
| fecal human cathe­licidin LL-37 level (pg/g) | | | | | |
| Baseline | median (P25, P75) | 6432.06(4508.38, 6822.43) | 5157.60(4557.45, 6003.06) |  |  |
|  | mean ± SD | 5823.35±1252.54 | 5281.84±995.48 | 1.55 | 0.1291 |
| After intervention | median (P25, P75) | 4523.09(3443.83, 5207.88) | 3236.43(3057.50, 4437.63) |  |  |
|  | mean ± SD | 4415.00±1036.93 | 3679.49±871.18 | 2.48 | 0.0175 |
|  |  | *t*=4.30, *p*<0.001^#^ | *t*=5.28, *p<*0.001^#^ |  |  |
| fecal sIgA level (μg/g) | | | | | |
| Baseline | median (P25, P75) | 89.93(77.60, 96.91) | 92.35(83.64, 107.68) |  |  |
|  | mean ± SD | 89.01±13.06 | 95.30±12.65 | 1.60 | 0.1165 |
| After intervention | median (P25, P75) | 83.43(76.97, 90.34) | 84.86(72.50, 96.06) |  |  |
|  | mean ± SD | 82.71±9.61 | 84.42±12.58 | 0.51 | 0.6148 |
|  |  | *t*=1.943, *p*=0.060^#^ | *t*=2.658, *p*=0.012^#^ |  |  |
| fecal calprotectin level (ng/g) | | | | | |
| Baseline | median (P25, P75) | 1100.72(939.66, 1263.24 ) | 1154.28(978.47, 1298.00) |  |  |
|  | mean ± SD | 1098.71±189.38 | 1156.94±170.57 | 1.05 | 0.298 |
| After intervention | median (P25, P75) | 942.80(843.17, 1064.68) | 1051.72(878.89, 1102.42) |  |  |
|  | mean ± SD | 946.65±153.85 | 978.82±165.22 | 0.66 | 0.5136 |
|  |  | *t*=3.116, *p*=0.003^#^ | *t*=3.269, *p*=0.002^#^ |  |  |

IG, intervention group; CG, control group; SD, standard deviation. *, *t*-test comparison between the two groups for data with normal distribution and similar variance; #, *t*-test comparison within each group for data with normal distribution and similar variance.

Supplementary material-5


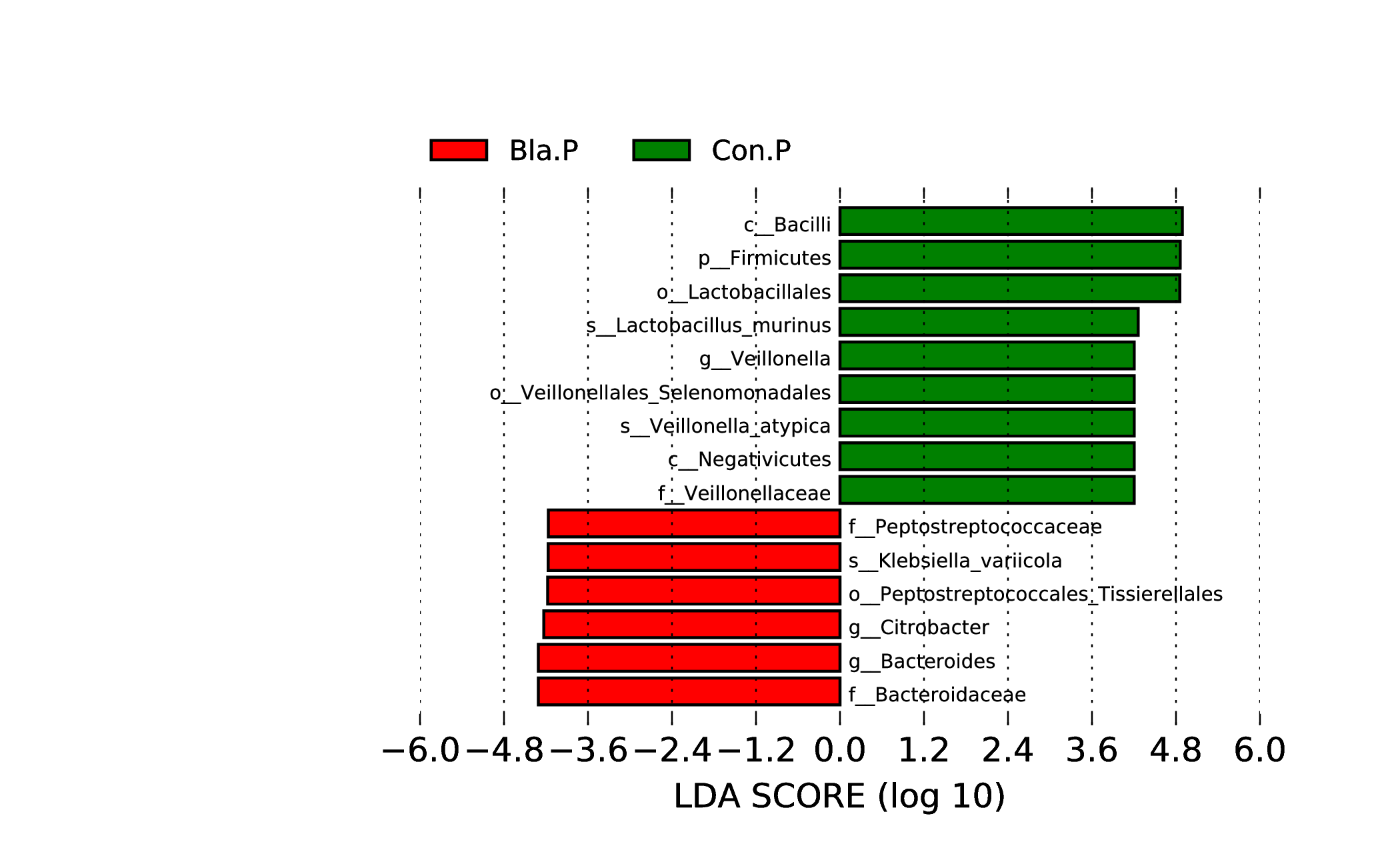


A


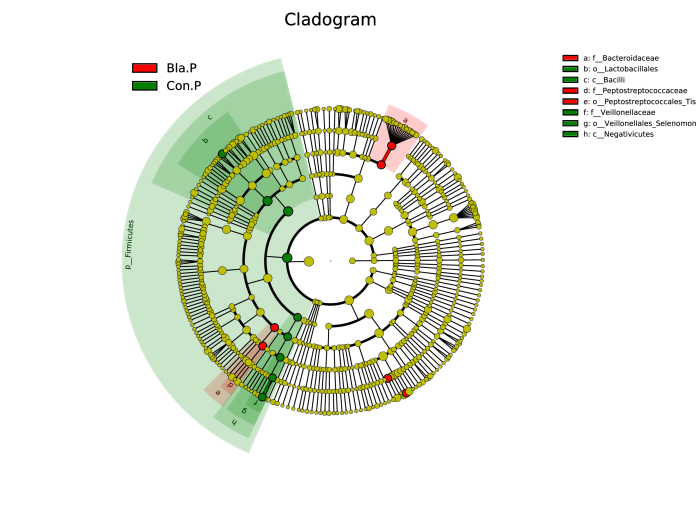


B


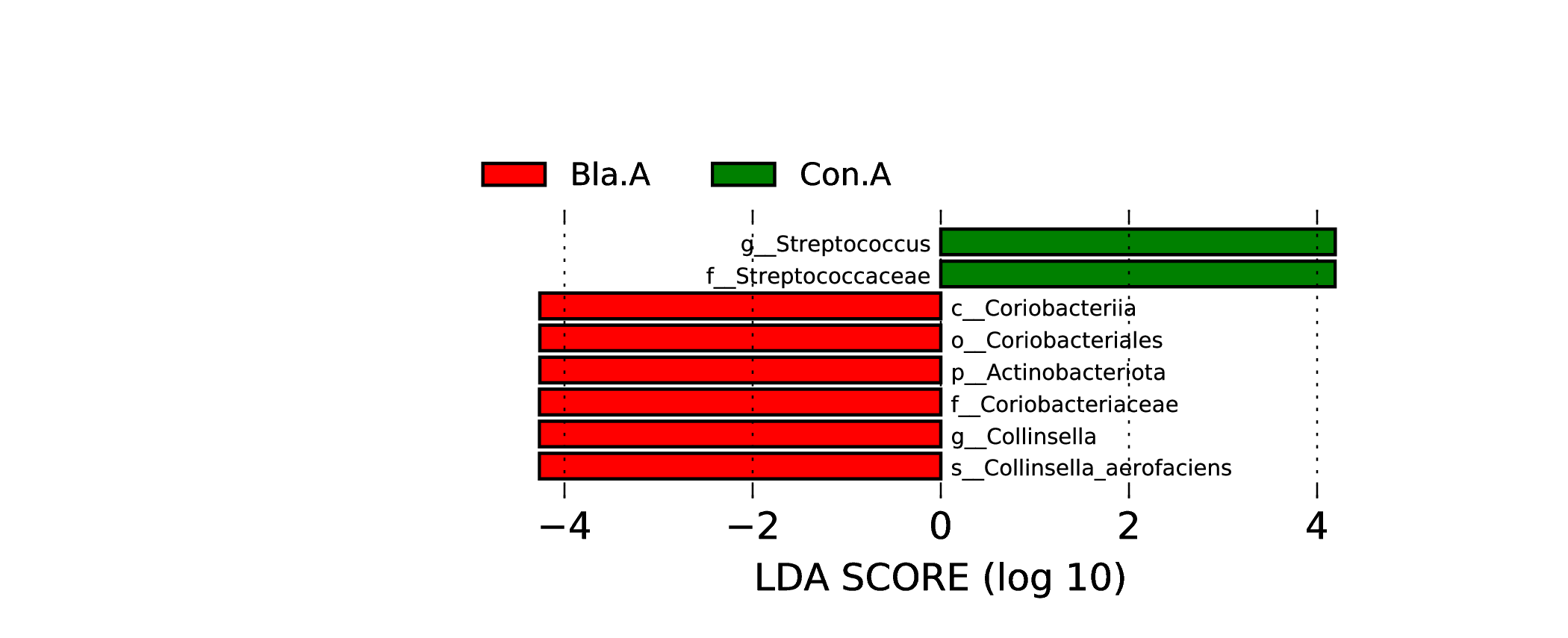


C


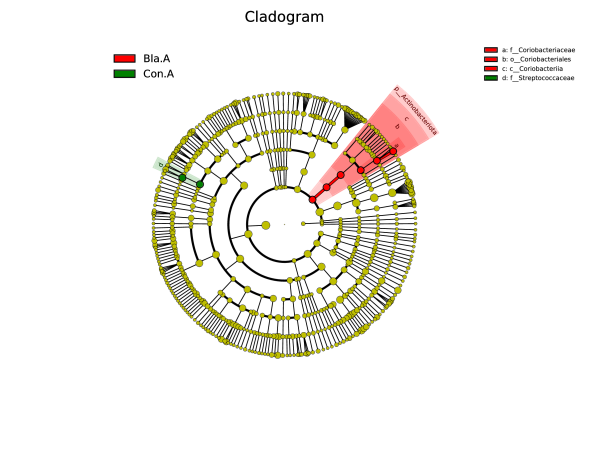


D

Figure 9: LEfSe analysis identified the most deferentially abundant taxa between the intervention and control groups. Cladogram: Taxonomic representation of statistically and biologically consistent differences among intestinal microbiota of different groups. Differences were represented by the color of the most abundant taxa (Green indicated a taxon with significantly higher relative abundance in the intervention group, red indicated a taxon significantly more abundant in the control group and yellow indicated no significant difference). LAD SCORE: Histogram of linear discriminant analysis (LDA) scores for deferentially abundant taxon. Cladogram was calculated by LefSe and displayed according to effect size. A, and B, LDA score and Cladogram before intervention; C and D, LDA score and Cladogram after intervention; Con.P, control group before intervention; Bla.P, intervention group before intervention; Con.A, control group after intervention; Bla.A, intervention group after intervention.

Supplementary material-6


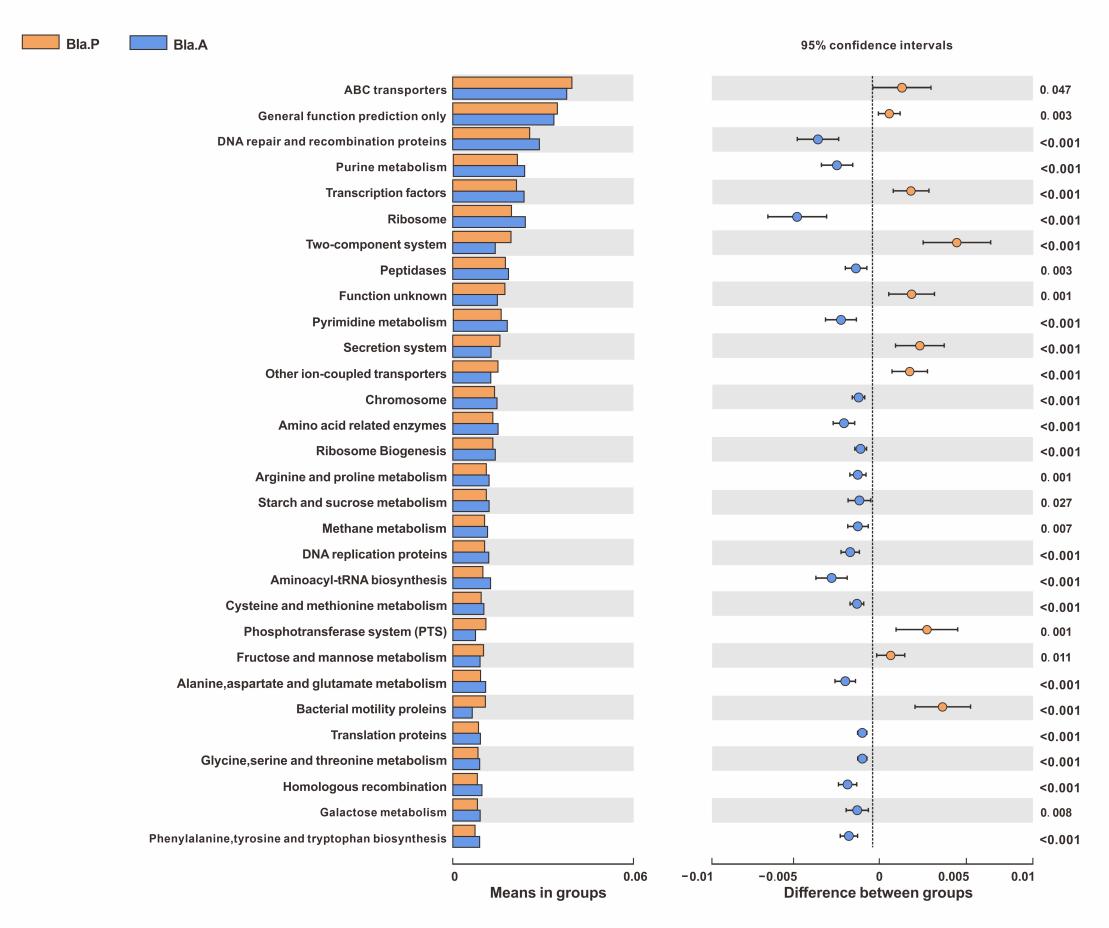


Figure PICRUSt function prediction of the gut microbiota in the BLa80 group with top 30 means in groups (Welch’s t test, two-sided, P<0.05) Bla.A, intervention group after intervention; Bla.P, intervention group before intervention.
